# Néphrectomie bilatérale de sauvetage compliquant une sclérose tubéreuse de Bourneville

**DOI:** 10.11604/pamj.2014.19.205.5483

**Published:** 2014-10-24

**Authors:** Mohamed El Amrani, Mounia Azizi

**Affiliations:** 1 Service de Néphrologie, Dialyse et Transplantation Rénale, Hôpital Militaire d'Instruction Mohammed V, Rabat, Maroc

**Keywords:** Sclérose tubéreuse de Bourneville, angiomyolipomes, kystes, reins, tubers, Bourneville tuberous sclerosis, angiomyolipoma, cyst, kidneys, tubers

## Image en medicine

La sclérose tubéreuse de Bourneville (STB) est une phacomatose autosomique dominante en rapport avec la mutation de deux gènes suppresseurs de tumeurs TSC1 et TSC2. L'atteinte cutanée et neurologique est constante, celle des reins ou de la rétine est fréquente. Une patiente âgée de 28 ans, suivie depuis l'enfance pour STB retenue devant l'association d'atteinte neurologique (tubers corticaux compliqués d'épilepsie), cutanée (tubers faciaux, tâches achromiques), hépatique, splénique et rénale (angiomyolipomes). La patiente fut admise aux urgences pour choc hémorragique objectivant à la TDM abdominale de volumineux angiomyolipomes rénaux bilatéraux spontanément hyperdenses évoquant un saignement intralésionel (A). Par ailleurs, on notait des angiomyolipomes hépatiques, un épanchement intrapéritonéal et une thrombose de la veine cave inférieure. Une embolisation de l'artère rénale était tentée sans succès (B). L'évolution était marquée par l'apparition d'un syndrome de compartiment avec une détresse respiratoire et hémodynamique ayant motivé une néphro-surrénalectomie bilatérale d'hémostase en urgence. Un traitement de suppléance de la fonction rénale est initié par hémodialyse dans l'attente d'une transplantation rénale, ainsi qu'une corticothérapie substitutive de la fonction surrénalienne. L'atteinte rénale doit être dépistée précocement et comprend des angiomyolipomes et/ou des kystes rénaux. La présence de plages de nécrose ou d'hémorragie doit faire éliminer un carcinome rénal. Les principaux diagnostics différentiels sont la neurofibromatose type 1 et 2 et la maladie de Von Hippel Lindau. Le pronostic dépend de la sévérité des symptômes et de leur évolution imprévisible. La recherche médicale est toujours d'actualité pour retarder au maximum la survenue de tumeurs.

**Figure 1:**
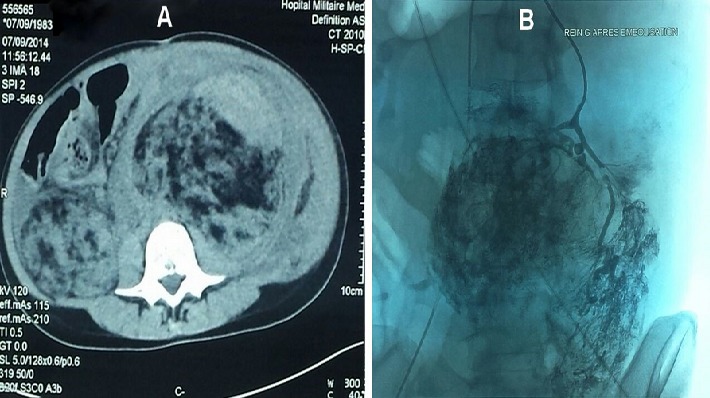
(A) Tomodensitométrie abdominale: volumineux angiomyolipomes rénaux bilatéraux spontanément hyperdenses; (B) Artériographie rénale: hypervascularisation rénale et diffusion extrarénale du produit de contraste

